# The Role of Predictability in Saccadic Eye Responses in the Suppression Head Impulse Test of Horizontal Semicircular Canal Function

**DOI:** 10.3389/fneur.2017.00536

**Published:** 2017-10-18

**Authors:** Jorge Rey-Martinez, Joaquin Yanes, Jonathan Esteban, Ricardo Sanz, Eduardo Martin-Sanz

**Affiliations:** ^1^ENT Unit ORLGipuzkoa, Hospital Quironsalud Donostia, San Sebastián, Spain; ^2^ENT Department, Hospital Universitario Donostia, San Sebastián, Spain; ^3^ENT Department, Hospital Universitario de Getafe, Getafe, Spain

**Keywords:** suppression head impulse paradigm, video head impulse test, vestibulo-ocular reflex, saccade, anticipation, preprogramation, early saccade

## Abstract

**Background:**

In the suppression head impulse paradigm (SHIMP) vHIT protocol, the participant is instructed to follow with his gaze a mobile target generated by a laser placed on the participant’s head. Recent studies have reported that the refixation saccade latencies are in relation with the time evolution of the vestibular dysfunction in both (standard and SHIMP) procedures. We hypothesized that some central mechanisms like head impulse prediction could be one of the causes for the differences in the saccadic eye responses.

**Methods:**

A prospective cohort non-randomized study was designed. For the SHIMP protocol, recorded with the ICS Impulse ver. 4.0^®^ (Otometrics A/S, Taastrup, Denmark) vHIT device, three different algorithms were performed: “predictable,” “less predictable,” and “unpredictable” depending on the target’s predictability. A mathematical method was developed to analyze the SHIMP responses. The method was implemented as an additional tool to the MATLAB open source script for the extended analysis of the vHIT responses named HITCal.

**Results:**

In cohort 1, 52 participants were included in “predictable” SHIMP protocol. In cohort 2, 60 patients were included for the “less predictable” and 35 patients for the “unpredictable” SHIMP protocol. The participants made more early saccades when instructed to perform the “predictable” paradigm compared with the “less predictable” paradigm (*p* < 0.001). The less predictable protocol did not reveal any significant difference when compared with the unpredictable protocol (*p* = 0.189). For the latency of the first saccade, there was statistical difference between the “unpredictable” and “predictable” protocols (*p* < 0.001) and between the “less predictable” and “predictable” protocols (*p* < 0.001). Finally, we did not find any relationship between the horizontal vestibulo-ocular reflex (hVOR) gain and the latency of the saccades.

**Conclusion:**

We developed a specific method to analyze and detect early SHIMP saccades. Our findings offer evidence regarding the influence of predictability on the latency of the SHIMP saccadic responses, suggesting that early saccades are probably caused by a conditioned response of the participant. The lack of relationship between the hVOR gain and the latency of the saccades suggests that the predictive behavior that caused the early eye saccades are independent of the vestibular function.

## Introduction

The suppression head impulse paradigm (SHIMP) was introduced in 2016 by MacDougall et al. (1) as an alternative paradigm to the conventional head impulse paradigm (HIMP) for the video head impulse test (vHIT).

The main difference between both paradigms is that during the HIMP protocol, short, fast, and unpredictable head turns are performed passively by the examiner on the head of the participant who is instructed to keep his/her gaze on a ground-fixed target placed in front of the participant (2), in the SHIMP protocol, the same impulses are performed by the examiner, but the participant is instructed to follow with his/her gaze a mobile target generated by a laser source placed on the participant’s head. This configuration enables the projected laser dot to move jointly with the participant’s head movement (1, 2).

When a healthy participant is tested with the HIMP procedure, a slow phase (3) eye movement commanded by the vestibulo-ocular reflex (VOR) opposed to the head movement enables the participant to maintain gaze on the ground fixed target. When a healthy participant is tested in the SHIMP protocol, after the slow phase movement, a saccadic movement (4) will correct the eye position to match it with the new position of the laser target that has been moved to a new position due to the participant’s head movement (2).

When a patient with vestibular function loss is tested with the HIMP protocol, the corrective saccade may be registered, but not when the same patient is tested with the SHIMP protocol (2).

Therefore, the refixation saccades derived from the complementary vHIT testing procedures are observed in different vestibular functional statuses. For example, the HIMP refixation saccades will appear in participants with vestibular impairments, but not in healthy participants, while the SHIMP saccades will appear in healthy participants, but not in those with vestibular impairments (1). However, not only the appearance of the refixation saccades but also the time of their appearance is different between the two paradigms. In the HIMP paradigm, the refixation saccades had been classified into the covert and overt saccades depending on the time of their appearance. A covert saccade will always begin during the participant’s head movement with a latency shorter than 80 ms, and an overt saccade will appear after the participant’s head movement has stopped with a latency exceeding 80 ms. The catch-up saccades are thought to be an indirect cognitively determined indicator of semicircular canal function (5).

In the bilateral vestibular impaired patient, their influence only appeared at approximately 70 ms after the onset of head rotation (6). In the SHIMP paradigm, the refixation saccades will appear mainly at approximately 80 ms after the head impulse, implying that the SHIMP corrective saccades will appear after the head movement.

Although the cause underlying the latency differences of the (compensatory and anticompensatory) corrective saccades in the latency periods between the HIMP and SHIMP procedures has not been well established, recent studies have reported that the refixation saccade latencies are in relation with the time evolution of the vestibular dysfunction in both procedures (5, 7). We hypothesized that some central mechanisms such as the head impulse prediction could be one of the causes behind the observed differences in the saccadic eye responses for the SHIMP paradigm.

In the present study, we developed a methodology to identify early saccadic responses defined as the SHIMP saccades that occur during the head movement. We also analyzed the presence and main characteristics of these early saccadic responses in participants according to three SHIMP protocols, namely one protocol designed as “unpredictable” and two designed as “predictable” and “less predictable” protocols.

## Materials and Methods

This section is divided into two parts, with the first part focusing on the development of a specific mathematical method to analyze the vHIT SHIMP responses and the second part focusing on the clinical study performed to characterize the SHIMP responses in participants according to different SHIMP testing procedures varying predictability paradigms.

### Mathematical Method to Analyze the SHIMP Responses

This method was developed to analyze the SHIMP responses recorded with the ICS Impulse ver. 4.0^®^ (Otometrics A/S, Taastrup, Denmark) vHIT device. The SHIMP analysis method was written using MATLAB (MATLAB Release 2015b macOS 64-bit version, The MathWorks, Inc., Natick, MA, USA). The method was implemented as an additional tool to the MATLAB open source script for the extended analysis of the vHIT responses named HITCal (8). The SHIMP analysis method and the HITCal MATLAB scripts have been both published as an open source software and can be downloaded from the HITCal GitHub repository (https://github.com/bendermh/HITCal).

#### Data Source

The SHIMP test responses were exported in Extensible Markup Language (XML) file format from the ICS Impulse^®^ manufacturer default database. The XML files where imported to HITCal to be analyzed with the SHIMP analysis module described in the next paragraphs.

#### SHIMP Responses Analysis

The eye and head velocity data were computed with HITCal. The first step of the analysis was to determine local maxima points and curves from the eye and head velocity data for each impulse by using the MATLAB signal processing toolbox “findpeaks” function. The head velocity data were analyzed once with this function, while the eye velocity data were analyzed twice with this function. The first analysis of the eye data was performed on the original data and the second analysis was performed by using the inverse velocity eye data. The eye velocity data were processed twice because the “findpeaks” function only recognizes the positive local maxima data and the SHIMP saccades are in the opposite directions to the slow eye response (VOR). With these two analyses of the eye velocity data, we obtained the VOR peak response in the first analysis and the (quick) saccadic eye responses in the second analysis.

**Figure 1:**
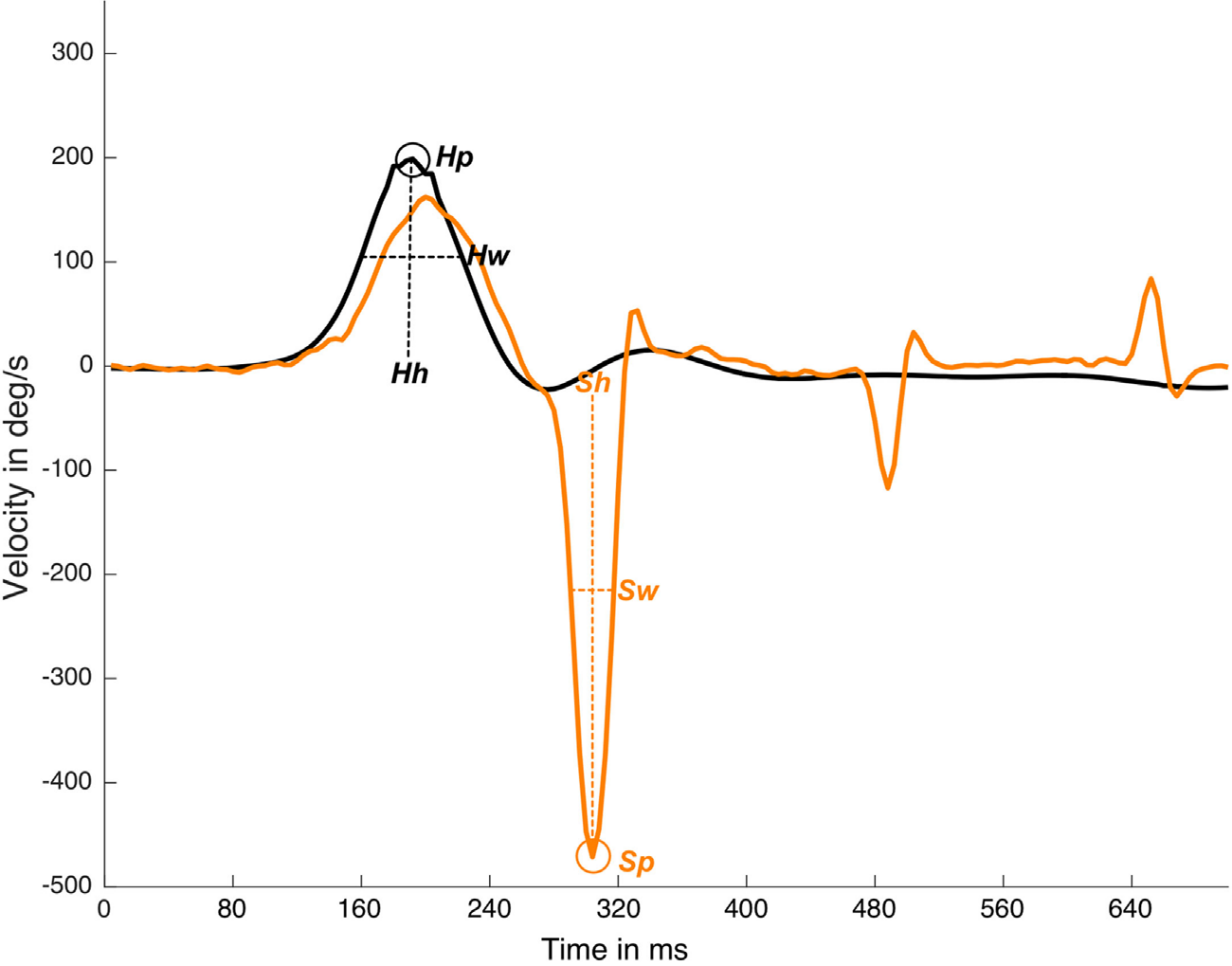
Detection and measurements of suppression head impulse paradigm (SHIMP) impulse and response plot. In this real data plot, one head peak was detected (Hp) and one saccadic eye response was detected (Sp). Response latency is defined by Sp time value less Hp time value, Hh is the head response height measured from the head curve baseline to head curve peak, Sh is saccade height also calculated for eye saccadic response. Hw is the head response weight measured as the curve weight at the half of the head curve height, Sw is saccade weight also calculated for eye saccadic response. *y*-axis is head and eye velocity in deg/s and *x*-axis is time in samples (with an ~250 Hz sampling frequency), black line is head velocity plot, orange line is eye velocity plot.

The MATLAB “findpeaks” function was computed with two arguments, “NPeaks” and “MinPeakProminence.” The “NPeaks” argument was set to the value of 1, and was used to specify that only one local maximal must be outputted for each velocity plot data. The “MinPeakProminence” argument was set to 100 for the head data and to 80 for the eye data. For these reference values to consider one local maxima as true, it is important to note that the peak “prominence” was used instead of the real maximum velocity on the peak value to detect a saccade, where prominence is the velocity value in reference to the basal eye or head velocity line, but not in relation to the *x*-axis line (Figure 1).

The “Findpeaks” function outputted for each (head and eye) plot of one local maxima peak were defined by the peak time position, peak real velocity value, peak width, and peak prominence (Figure 1).

#### Early SHIMP Saccades Identification

**Figure 2:**
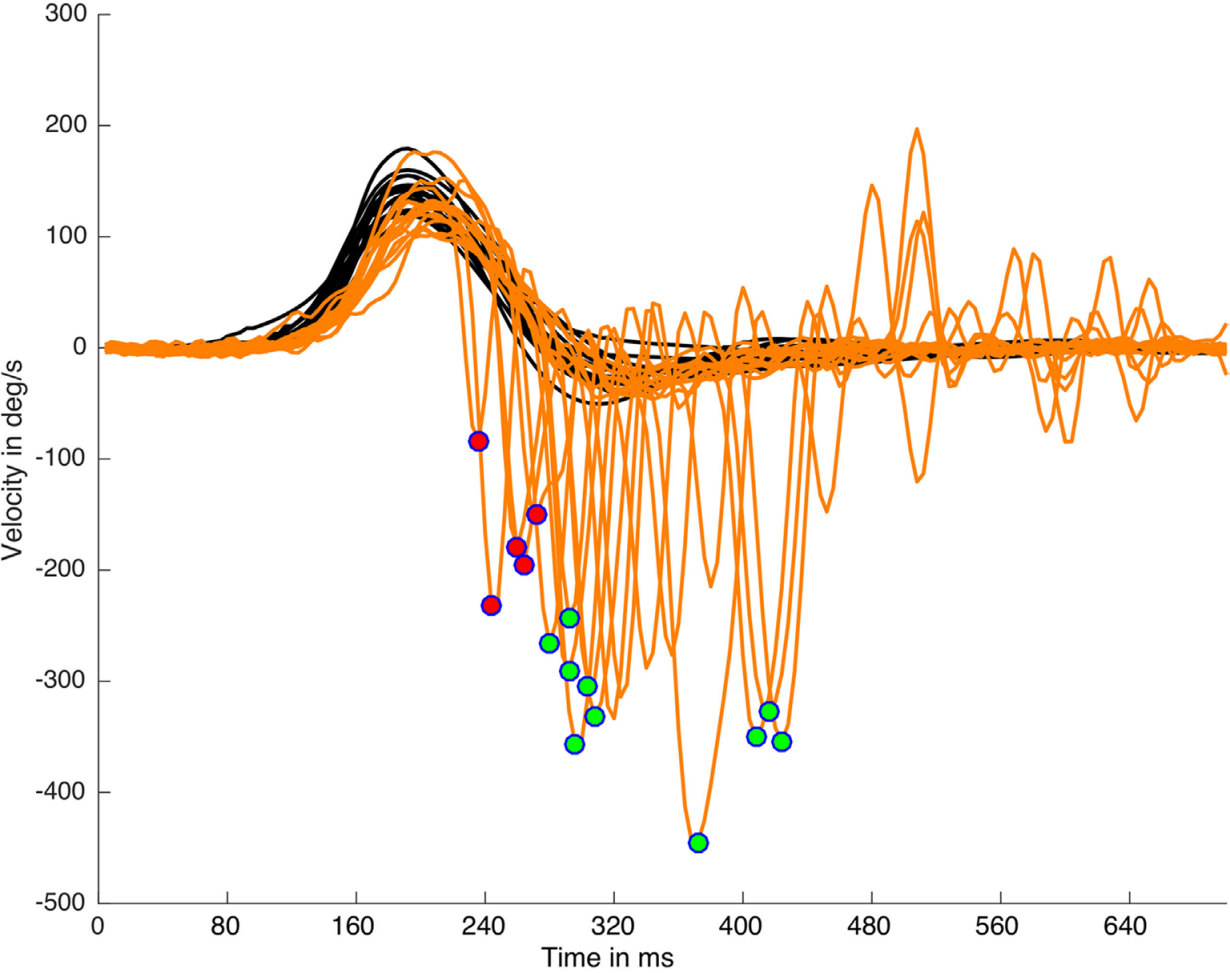
Suppression head impulse paradigm (SHIMP) saccades detection and classification on a real data (left side) plot processed with HITCal. After the specific SHIMP saccades detection an algorithm based on head impulse weight classified these saccades as early SHIMP saccades (marked with red circles) or not early SHIMP saccades (marked with green circles) depending if they exceed or not exceed the head impulse time window. Only one saccade is detected and classified per each head impulse. Black lines are head impulses, orange lines are eye responses.

With the obtained parameters, an algorithm was developed to identify the early SHIMP saccades. An early SHIMP saccade was identified whether its time of appearance was less than the early saccade time period value (ESTP); ESTP was obtained from this formula:

$$\text{ESTP}=\text{Hpt}+\frac{\text{Hw}}{\text{1.5}}+\text{Sw,}$$

where Hpt is the head velocity peak time appearance, Hw is the head impulse width, and Sw is the saccade width (Figure 1). This algorithm was able to detect early SHIMP saccades (Figure 2) independently of the return to the 0 value parameter of the head velocity plot and was based on the morphology of the head impulse and the eye saccadic responses.

The method used to detect the early saccades was based on a time period determined by the duration of the head impulse and the first saccadic eye response. This time period was calculated using the width on both the eye and head responses at the half width of half height of the curves prominence. This new width-based method is an alternative to other methods, such as the covert time period for the HIMP conventional vHIT protocol where the time period for the covert responses is defined by the time interval from the peak of the head response to the moment when the head velocity crosses the 0°/s value in the *x*-axis. This and other similar methods for head impulse responses characterization based on time intervals determined by fixed velocity values (5) were considered for this study to calculate the early eye responses.

**Figure 3:**
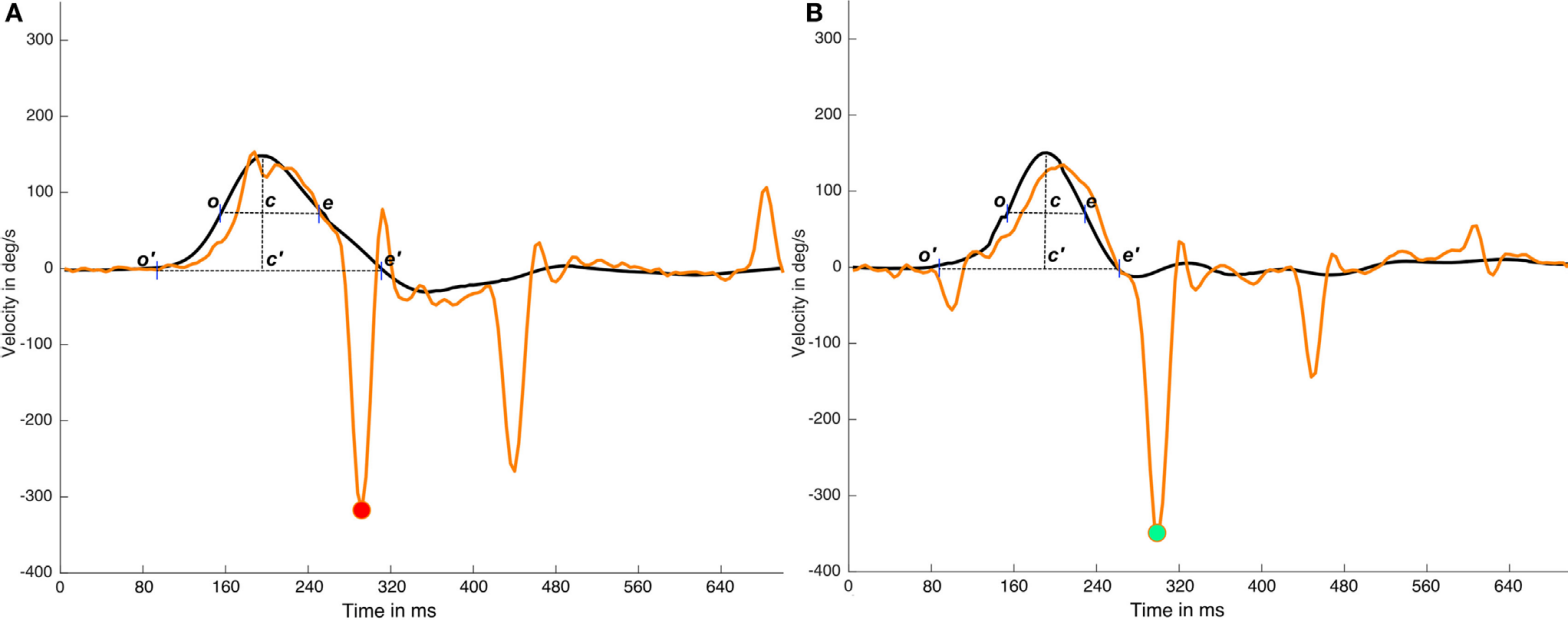
Effects of head impulse asymmetries on time period calculations. In this figure, two impulses (real data) from the same video head impulse test (vHIT) test are plotted; in the **(A)** impulse it is plotted an impulse with a very large and asymmetric head impulse (c′e′ > o′c′) with a cross point on *x*-axis (e′) that occurs at ~80 samples time, in **(B)** impulse it is plotted an impulse with a more symmetric head impulse (c′e′ ≈ o′c ′). **(A)** and **(B)** has a first saccadic response at the same time moment ~70 samples. If we consider the c′e′ time interval, determined by the cross to 0°/sec point (e′) to calculate the early response period, the same saccadic response is classified as early on **(A)** impulse and as not early on **(B)** impulse. But if the width of the head impulse (ce segment in plots) is used to calculate the early response period, because it is less affected by the head impulse asymmetry, the first saccade will be recognized as not-early on both **(A)** and **(B)** impulses. Black lines are head impulses, orange lines are eye responses, magenta point is marking the (wrong) early eye saccade on the **(A)** impulse and green point is marking the not early saccade in the **(B)** impulse.

Under ideal conditions, these classical methods are an easy way to determine the time periods in head impulses. However, the return to a velocity value of 0 is determined by the quality and the shape of the performed head impulses, indicating that, in some head impulses, this return to 0 point is influenced by the head movements that occur after the original head impulse. The overshoot of the head impulse (5) is a well-known phenomenon that occurs with a variable intensity in most of head impulses. Overshoot and other asymmetric head movements that occur on some head impulses could have a significant influence on the time period calculation (Figure 3). Based on these observations, the alternative method described here was designed to be impervious to the effects of asymmetric head impulses in the time period calculation.

**Figure 4:**
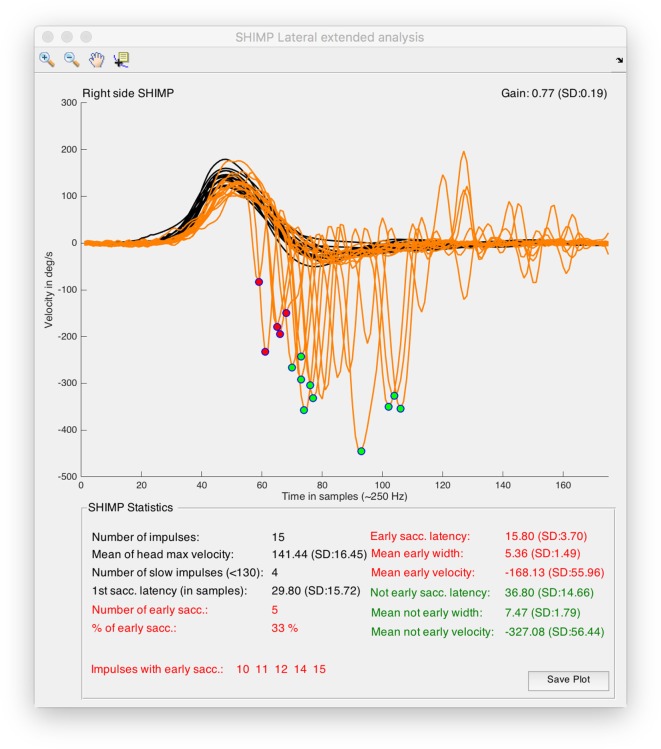
Output window obtained from suppression head impulse paradigm (SHIMP) analysis tool of HITCal running on macOS™ High Sierra™ computer. Real SHIMP data (left side) is computed, on the superior half of the window SHIMP is plotted with the detected and classified saccades were marked with red and green circles. On the top of the plot there are the plot navigation tools, include a data cursor view to allow manual measurement of the plots. On the inferior half of the window statistical data from SHIMP test analysis is outputted, also in this part red color are used for the computed results of the detected early SHIMP saccades, and green color are used for the computed results of the detected not early SHIMP saccades, black color is used for the head impulse analysis and also for the first saccade analysis, where first saccades are all the first detected saccades for each head impulse computing both the early and not early classified saccades.

For all the computed data, simple arithmetic algorithms were written to calculate these other parameters including the number of impulses per SHIMP test, mean of the head peak velocity values, number of head impulses with peak velocity under 130°/s, first SHIMP saccade latency, number and percentage of early SHIMP saccades, list of impulses with latency early SHIMP saccades, early and not-early SHIMP saccades latency, width, and velocity (Figures 1, 2 and 4).

### Clinical Study Design and Development

#### HIMP and SHIMP VHIT Protocols Procedures

For this clinical study, the HIMP test was performed on each participant using the standard HIMP test protocol as described by Cuthoys et al. (9). Fast, short, and unpredictable head impulses were performed in random horizontal directions while the participant was seated in front of a ground fixed target and was instructed to maintain his/her vision continually fixed on the target during the HIMP test.

For the SHIMP protocol, three different methods based on the HIMP protocol as was originally described (1) were performed in this study:

– “Predictable” SHIMP protocol.
– “Less predictable” SHIMP protocol.
– “Unpredictable” SHIMP protocol.

In the predictable SHIMP protocols, a fast and short head impulse, without an objective randomized side selection, together with a rhythmic and fast repetition of impulses, was performed. The participant was only instructed to follow a laser light spot projected from a head-mounted device on the participant’s head; thus, this laser spot followed the participant’s head movement. In order to analyze the possible differences depending on the predictability, two different cohorts were used to test this easily predictable method. In one of the two cohorts, the examiner performed consecutive right and left impulses after announcing the direction of the next impulse in a loud voice, with an inter-impulse interval close to 1 sec, so that the participant could anticipate the direction of the next impulse in a true conditioned way; this method was also named the “predictable” protocol. In the other variant of this method, the tested participants were not verbally informed about the direction of the next head impulse. This second “less predictable” protocol was designed to allow to the participant to predict the procedure easily, thus avoiding the possible conditioned responses derived from the verbal indication of the examiner.

The third “unpredictable” SHIMP protocol was performed by using fast and brief head impulses. The impulses were performed to avoid any predictive clue for the tested participant. Therefore, the examiner was instructed to perform fast head impulses with slow velocity movements while returning the participant’s head after the head impulse to the straight head position. After each impulse, a long-time pause (>5 s) was performed while the examiner held the participant’s head in a straight position. Furthermore, the participant was instructed to follow the laser spot without any attempt to predict or to anticipate the head movement. During this third “unpredictable” protocol, all the tested participants were never informed about the direction of the next head impulse.

#### Clinical Study

A prospective cohort non-randomized study was designed to evaluate the main hypothesis of this article. Consecutive participants were examined by two senior vHIT examiners from two reference medical centers between June 2016 and May 2017. The examiners were instructed to explore the participants following the previously described protocols. Visual impairments, neurological impairments, and inability to understand and/or follow the examiner’s instructions where the exclusion criteria for this study.

In the first center, one cohort of 56 participants was included. The vHIT “predictable” SHIMP protocol was performed in this cohort, as described earlier, whereby the patients of the easy-to-predict protocol received an anticipatory oral information by the examiner regarding the direction of the next impulse.

In the second center, two cohorts were included. In one cohort, 60 participants were examined with the “less predictable” SHIMP protocol that was performed in this case without any oral indication given by the examiner about the direction of the next impulse, as was described earlier. This method was easy to predict due to its simple and repetitive rhythmic sequence. A second cohort of 35 participants was also examined with the SHIMP “unpredictable” protocol.

The three cohorts were classified with either a normal horizontal vestibulo-ocular reflex (hVOR) function or an altered hVOR function according to the values adjusted by age and sex previously published (10, 11).

In addition to the methods integrated into the device by the manufacturer to ensure adequate head impulses records, all the impulses were reviewed to verify that a minimum head impulse velocity of 120°/s was reached for each impulse and also all the impulses were reviewed to ensure that there were no irregularities in the eye and head velocity plots.

Written informed consent was obtained from all participants. Because the nature of the intervention was not new or exceptional, only the approval of the local ethical committee in each corresponding center was required for all clinical researchers. The study was designed and performed in accordance with the ethical guidelines of the 1975 Declaration of Helsinki.

All vHIT tests were performed with the ICS impulse^®^ ver. 4 (Otometrics A/S, Taastrup, Denmark) vHIT device.

#### Statistical Analysis

All statistical analysis was performed using SPSS (IBM SPSS Statistics for macOS, Version 22.0., IBM Corp., Armonk, NY, USA) and MATLAB (MATLAB Release 2015b, The MathWorks, Inc., Natick, MA, USA) with statistical toolbox.

When analyzing the relationship between the type of participants and the different protocols, we conducted a repeated measures analysis of variance (ANOVA) with the type of participants (healthy/patients) and paradigm type (predictable/less-predictable/unpredictable) as within-subject independent variables.

A multivariate ANOVA (MANOVA) was conducted with the cohorts as independent variables, and with the head velocity, latency, width, and velocity of the saccades as dependent variable, when studying such variables.

We performed a linear regression when analyzing the relationship between the hVOR gain and the percentage of early saccades.

For the results presented in this manuscript, a constant sampling rate of 250 Hz was assumed for the time calculation in milliseconds.

## Results

### Demographics

In cohort 1, 52 participants were included and tested according to the “predictable” SHIMP protocol. Of those, 27 were healthy participants (mean age 50.59 ± 3.16 years; range 24–90 years) and 25 were patients with unilateral vestibular loss (UVL) (mean age 48.44 ± 2.65 years; range 19–81 years). No significant differences in the age and sex distribution were observed between both groups.

The mean time from onset of the UVL was 12.6 ± 0.17 months (range 11–14 months). There was a significant difference (*p* < 0.05) in the mean hVOR gain when both healthy participants and patients were compared. The mean hVOR gain was 0.43 ± 0.03 (range 0.21–0.69) in the affected ear and 0.86 ± 0.45 (range 0.68–1.01) in the healthy ear.

In cohort 2, 95 participants were included. The “less predictable” SHIMP protocol was performed in 60 participants (mean age 55.95 ± 2.14 years; range 16–88 years), and the “unpredictable” SHIMP protocol was performed in 35 participants (mean age 52.26 ± 3.03 years; range: 17–90 years). No significant differences in the age and sex distribution were observed between both groups.

The mean hVOR gain was 1.02 ± 0.01 (range 0.51–1.33) for the right ear and 0.89 ± 0.12 (range 0.48–1.20) for the left ear.

### Percentage of Early Saccades

**Figure 5:**
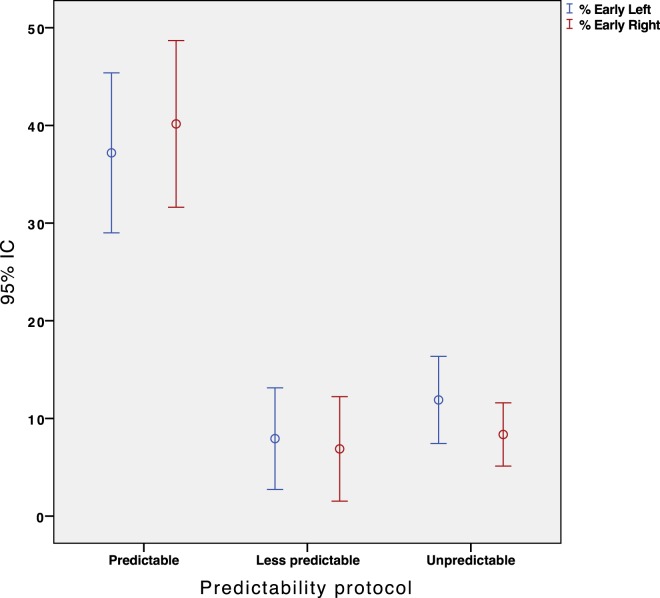
Error Bar’s graph representing the percentage of early eye saccades obtained in our population depending on the protocol performed. Red and blue color represent the right and left sided impulses, respectively.

We conducted a repeated measures ANOVA with the type of participant (healthy/patients) and paradigm type (predictable/less-predictable/unpredictable) as within-subject independent variables. The analysis yielded a significant main effect for the predictability type, *F*(3, 146) = 36.25, *p* = 0.001. Overall, the participants made significantly more early saccades when instructed to perform the “predictable” paradigm compared with the “less predictable” paradigm (*p* < 0.001, Figure 5). Nevertheless, the less predictable protocol did not reveal any significant difference in the percentage of early saccades when compared with the unpredictable protocol (*p* = 0.189, Figure 5).

**Figure 6:**
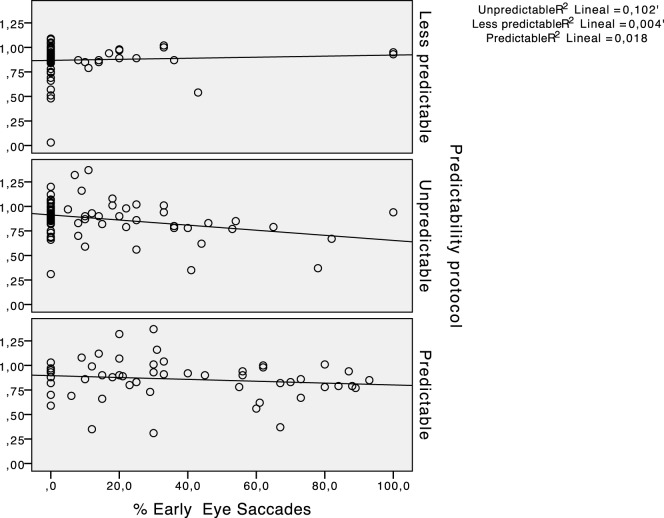
Scatter plots representing the relationship between horizontal vestibulo-ocular reflex (hVOR) gain and the percentage of early saccades in every predictability protocol performed.

The results for the type of participant did not show any main effect for early saccades (*F* = 1.356, *p* = 0.247). The percentage of early saccades was significantly higher (*p* < 0.05) in the predictable paradigm regardless of the participants group. Figure 6 shows the absence of a statistically significant relationship between the hVOR gain and the percentage of early saccades.

The values for latency, velocity, and width of early saccades are summarized in Table 1. The mean latency of early saccades was 62.96 ± 4.28 and 78.72 ± 7.76 ms for the “predictable” and “unpredictable” protocols, respectively. The comparison between the latencies of the early saccades observed in both paradigms showed no significant differences (*p* = 0.21). In the same way, the analysis did not reveal any significant differences (*p* > 0.05) in the width of the early saccades when the “less predictable” paradigm was performed compared with the “predictable” paradigm. In summary, when the early eye saccades were present, they were similar in latencies, width, and velocity.

**Table 1:** Mean of measurements for early and first saccades obtained with the different predictability groups.

Early saccades	Predictability protocol		
	Predictable	Less predictable	Unpredictable
Percentage (%)	40.2 ± 4.2	7.9 ± 7.2	8.4 ± 5.8
Latency (ms)	62.96 ± 4.28	78.72 ± 7.76	66.68 ± 8.56
Width (ms)	26.56 ± 1.48	38.36 ± 4.48	23.56 ± 3.2
Velocity (deg/s)	242.07 ± 12.50	220.58 ± 26.02	286.00 ± 42.33
**1st saccades**			
Latency (ms)	112.8 ± 56.96	152.04 ± 58.00	178.32 ± 46.04

### Cohorts

A MANOVA was conducted with the cohorts as independent variables, and the head velocity, latency, width, and velocity of the saccades as dependent variables. The analysis did not elicit any significant differences in the values of the latency, velocity, and width of the saccades between both cohorts in either of the two algorithms. Nevertheless, the head velocity during the produced impulse was significantly higher (*p* < 0.05) in cohort 1 than in cohort 2.

Both cohorts showed a higher percentage of early saccades when the “less predictable” algorithm was performed, compared with the more predictable protocol. In cohort 1, such percentage of early saccades was significantly higher (*p* < 0.05) in both algorithms than in cohort 2.

### Latency of the First Saccadic Responses

**Figure 7:**
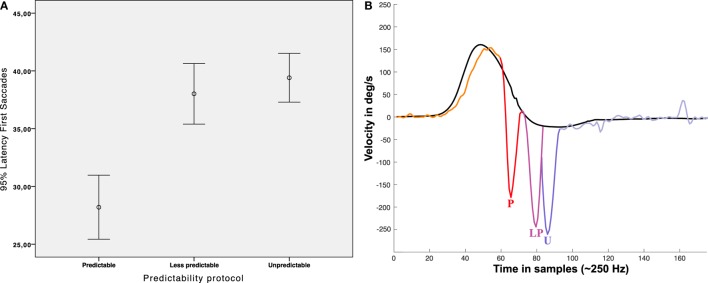
Latency of first saccades. **(A)** Error Bar’s graph representing the latency of the first saccadic response obtained in our population depending on the protocol performed. **(B)** Infographic plot showing nearest to mean measured first saccades latency for each protocol; P: (mean) early saccade obtained with predictable protocol (~112 ms), LP: (mean) Not early saccade obtained with less predictable protocol (~154 ms), U: not early saccade obtained with unpredictable protocol (~178 ms), all the mean latencies were statistical significant (*p* < 0.05). In **(B)**, head impulse (black line) and eye responses (multicolor line) are approximated scaled plots obtained from real data plots.

The measured mean latency of the first saccadic (early or not early) responses (measured from onset of head velocity) was 178.32 ± 46.04 ms for the “unpredictable” protocol, 152.04 ± 58.00 ms for the “less predictable” protocol, and 112.8 ± 56.96 ms for the “predictable” protocol (Figure 7, Table 1). Univariate ANOVA was performed with the latency as independent variable and the three SHIMP protocols as dependent variables and revealed statistical significance (*p* < 0.001). The *post hoc* Dunnett’s test showed a measured difference between the “unpredictable” and “less predictable” protocols of 26.24 ms, which was statistically significant (*p* < 0.001). The measured difference was 65.52 ms between the “unpredictable” and “predictable” protocols (*p* < 0.001) and 39.24 ms between the “less predictable” and “predictable” protocols (*p* < 0.001) by using the Bonferroni *post hoc* test. So as predictability increased the saccade latency decreased.

## Discussion

In this study, we developed a specific method to analyze and detect early SHIMP saccades to test the hypothesis that the appearance of these early saccades is associated with predictability. As described in the material and methods section, this new method was developed by using a new algorithm with one main difference from other methods of vHIT analysis (2), whereby the width of the saccades can be now measured and used to compute the early response time period (Figure 3). With this method based on the width of the stimulus and responses, we also developed a new method to define the early saccades time period (Figure 3). According to the results, we did not find evidence of significant error with this new classification method. For the latency of the early saccades, we observed a narrow standard deviation of 6.86 ms for an early saccades latency mean of 69.65 ms. In contrast, in the not-early saccades, we observed a wider standard deviation of 49.18 ms for a not-early saccade mean of 164.98 ms. These discrepancies could be an objective indicator of an adequate time period definition for early saccades. The statistical analysis did not show significant differences in the parameters (latency, velocity, and width) measured during early saccades in the three protocols. This uniformity supports the utility of the new developed classification method and suggests that the early saccades present similar and consistent properties on all the tested SHIMP protocols.

Two key features of sensorimotor prediction are preprogramming and adjusting of performance based on previous experience. The predictive behavior is based on the internal representation of target timing; therefore, the timing behavior is stored in the neural memory and is partly independent of the stimulus (12).

In the design of the SHIMP methods to evaluate the predictability, we had developed three strategies: one unpredictable and two predictable. The two predictable methods were developed with the aim to evaluate the possible slight but *a priori* significant differences in the predictability. In the “predictable” protocol, the participant was advised about the direction of the next head impulse; this method was developed to condition the anticipation in the patient’s responses. In the “less predictable” protocol, the participant was not informed about the direction of the next impulse, but the test timing and repetition allowed an easy anticipation by the participant. Finally, a hardest to predict and anticipate protocol (unpredictable) was developed and was carefully designed to avoid the participant’s predictability, consisting of a pseudorandom procedure with a brief and fast head impulse followed by a slow velocity to return to the initial position movement, which was performed each time. Furthermore, after each head impulse, the examiner in this protocol waited for a variable time period with a minimum pause of 5 sec to ensure an easy-to-predict timing during the test.

The results obtained with these SHIMP protocols indicated a significant increase in the percentage of early saccades (approximately from 8 to 40%), which occurred mainly during the head movement, only on the predictable protocol. Both predictable protocols encouraged the estimation of the time interval between the impulses, allowing the internal representation of the target timing (i.e., an internal clock). This suggested that early saccades are probably caused by a conditioned response of the participant.

Although none of the protocols could totally avoid these early saccades, the appearance was lower (<10%) in the protocols that did not include the examiner’s verbal indication of the direction of the next impulse. This may suggest that the verbal indication alone affected the subsequent timing behavior through the feedback of recent past consecutive head impulses.

Apart from the SHIMP protocol, none of the other variables considered in this study (e.g., hVOR gain) showed a statistical significance with the percentage of early saccades.

Although the SHIMP saccades are thought to be absent in patients with vestibular impairments, we did not find any relationship between the hVOR gain and the latency of the saccades, suggesting that the predictive behaviors that caused the early eye saccades are independent of the vestibular function. The central programming of the eye movements contributes to the gaze stability during predictable head movements in both healthy persons and patients with vestibular dysfunction (13).

Saccadic responses measured in the SHIMP test have been recently reported to be associated with vestibular hypofunction and vestibular compensation (5). It has also been reported that non-vestibular inputs can trigger the saccadic response (14) observed in the SHIMP tests. Whether other mechanisms, such as the pursuit/optokinetic system or the cervico-ocular reflex, could generate compensatory eye movements that parallel the VOR may be controversial, but probably irrelevant in our paradigm that only analyzes the volitional saccades after a SHIMP protocol.

Although they have not been measured in this study, early saccades such as intrusions on the slow phase of the eye response (Figure 7) could have a significant impact on the VOR gain calculation, similarly to what occurs with unremoved covert saccades in the HIMP vHIT procedure (2, 15). However, it is not usual that the examiner gives a previous instruction to the participant according to the original description of the SHIMP test procedure (1). Therefore, the risk of a high incidence of early saccades is low with an adequate exploration technique. Thus, the interesting finding in the SHIMP test indicated a strong relationship between the certain predictability and the latency of the eye response.

In the cases of less predictable and unpredictable protocols, there was a significant short delay on the saccadic responses of the unpredictable protocol in comparison with the less predictable protocol (+26.24 ms), although there was no difference in the occurrence of early saccades (~8%). As mentioned earlier, the participants in both protocols were never advised about the direction of the next impulse. Indeed, the only difference between the two protocols was that the “less predictable” test had an easy-to-predict performance. This suggests that this time delay of the saccadic response is mainly depending on the (non-conditioned) predictability of the impulse. This relation between the predictability and the latency delay of the saccadic responses has been previously reported in head rotation based tests with a measured delay of 15 ms of eye responses as an effect of predictability (16). In the present study, we further presented the forced predictability as a mechanism that can influence the time latency of the saccadic responses.

In conclusion, the present findings offered a strong evidence regarding the influence of predictability on the latency of the SHIMP saccadic responses for both normal participants and participants with pathologic conditions. Despite the fact that there was an influence of predictability on the saccadic latency for the SHIMP procedure only in the case of verbal conditioned test procedures, this effect could affect the main outcome of the test. However, the effect of other less evident predictable procedures must be carefully considered when evaluating the responses of the SHIMP test procedure. In light of our results, we recommend making the head impulses as unpredictable and brief as possible.

## Ethics Statement

Written informed consent was obtained for all patients. Because of no new or exceptional interventions, only local ethical committee approval in each corresponding center was required for all clinical researchers. The study was designed and performed in accordance with the ethical guidelines of the 1975 Declaration of Helsinki.

## Author Contributions

JR-M and EM-S have designed this study, performed the data collection and written this article, JR-M has designed and developed of the SHIMP analysis method. JY, JE, and RS have contributed to data collection and the writing of this article.

## Conflict of Interest Statement

The authors report no conflicts of interest. The authors alone are responsible for the content and writing of the paper.
